# High CD44 Immunoexpression Correlates with Poor Overall Survival: Assessing the Role of Cancer Stem Cell Markers in Oral Squamous Cell Carcinoma Patients from the High-Risk Population of Pakistan

**DOI:** 10.1155/2022/9990489

**Published:** 2022-03-07

**Authors:** Yumna Adnan, S. M. Adnan Ali, Hasnain A. Farooqui, Hammad A. Kayani, Romana Idrees, M. Sohail Awan

**Affiliations:** ^1^Office of Academia and Research in Surgery, Department of Surgery, Aga Khan University Hospital, Karachi, Pakistan; ^2^Department of Biosciences, Faculty of Life Sciences, Shaheed Zulfikar Ali Bhutto Institute of Science and Technology, Karachi, Pakistan; ^3^Section of Histopathology, Department of Pathology and Laboratory Medicine, Aga Khan University Hospital, Karachi, Pakistan; ^4^Section of Otolaryngology, Head and Neck Surgery, Department of Surgery, Aga Khan University Hospital, Karachi, Pakistan

## Abstract

Oral squamous cell carcinoma (OSCC) is a top-ranked cancer in the Pakistani population, and patient survival has remained unchanged at ∼50% for several decades. Recent advances have claimed that a subset of tumour cells, called cancer stem cells (CSCs), are responsible for tumour progression, treatment resistance, and metastasis, which leads to a poor prognosis. This study investigated the impact of CSC markers expression on overall survival (OS) and disease-free survival (DFS) of OSCC patients. *Materials and Methods*. Immunohistochemistry was used to evaluate CD44, CD133, L1CAM, and SOX2 expression in a well-characterized cohort of 100 Pakistani patients with primary treatment naïve OSCC. The immunoreactivity for each marker was correlated with patient clinicopathologic characteristics, oral cancer risk chewing habits, and survival. The minimum follow-up time for all patients was five years, and survival estimates were calculated using the Kaplan–Meier method and Cox proportional hazards model. *Results*. In this cohort of 100 patients, there were 57 males and 43 females. The median OS and DFS time durations observed were 64 and 52.5 months, respectively. Positive expression for CD44, CD133, L1CAM, and SOX2 was observed in 33%, 23%, 41%, and 63% of patients. High CD44 expression correlated with decreased OS (*P*=0.047) but did not influence DFS. However, CD133, L1CAM, and SOX2 had no effect on either OS or DFS. Tonsils, nodal involvement, and AJCC stage were independent predictors of worse OS and DFS both. *Conclusion*. Of the CSC markers investigated here, only CD44 was a predictor for poor OS. CD44 was also associated with advanced AJCC and T stages. Interestingly, CD133 was significantly lower in patients who habitually consumed oral cancer risk factors.

## 1. Introduction

Oral cavity cancer is one of the leading causes of cancer-related death in South Central Asia, including Pakistan. It is the first and second most common cancer in Pakistani males and females, respectively, and has the second-highest rate of oral cavity cancers worldwide, thus continuing to be a major public health crisis and a significant hurdle in improving life expectancy [1, 2].

The rationale for the high incidence of oral cavity cancers in Pakistan, and South Asia in general, is the frequent, persistent, and prevalent use of substances classified as oral cancer risk factors. These include betel quid, areca nut, alcohol, smoking, and smokeless tobacco.

Despite recent advances in imaging technology and treatment modalities, the last few decades have seen limited improvement in the survival rate of oral cancer. At our centre, we have observed approximately 40–50% of patients survive five years following diagnosis [3].

More than 90% of oral cancers are oral squamous cell carcinomas (OSCC), arising from the squamous epithelia of the oral cavity. The cancer stem cell hypothesis states that cancer stem cells (CSCs) are a subpopulation of multipotent cells at the core of a tumour that is responsible for tumour differentiation, tumour maintenance, and spread to other sites [4]. CSCs are believed to evade or be resistant to conventional treatment and thus can generate new tumour cells that are genetically identical to the parent tumour. This self-renewal ability of CSCs leads to disease recurrence and treatment failure. The role of CSCs has not been fully elucidated in OSCC [5].

It may be that subpopulations of CSCs at the core of OSCC tumours are the source of tumour regrowth. To improve patient survival, there is a need to design therapies targeted towards identifying and eradicating this subpopulation of self-renewing cells. The identification of CSCs is made easier by detecting the increased expression of a panel of CSC markers present on their surfaces and within. Such CSCs markers include CD44, CD133, L1CAM, and SOX2.

CD44 is a cell surface glycoprotein that regulates cell proliferation, adhesion, migration, and invasion in CSCs. Increased CD44 expression has been noted in multiple cancers such as pancreas, stomach, colon, lung, breast, prostate, salivary glands, and head and neck, among others, and has been linked to worse prognosis [6]. In OSCC, the role of CD44 in predicting prognosis is debatable as conflicting results have been reported [7, 8].

Similarly, CD133 (also known as Prominin-1) is another cell surface glycoprotein identified in hematopoietic and progenitor cells. CD133 is responsible for growth, differentiation, and cell motility and is believed to cause tumour relapse and progression towards malignancy. It has been investigated as a possible prognostic factor for melanoma, thyroid carcinoma, prostate carcinoma, retinoblastoma, brain tumours, leukaemia, renal tumours pancreatic tumours, and oral cancer [9, 10]. However, in the case of OSCC, the prognostic impact of CD133 has not been fully validated as conflicting evidence exists.

Another factor that is critical for the maintenance and self-regeneration of stem cells is Sox2. Sox2 is a transcription factor modulating the expression of several genes essential for the maintenance of the embryonic stem cell phenotype. In cancer, Sox2 protein expression has been linked with a worse prognosis as it promotes drug resistance, metastasis, survival, and proliferation [11]. For OSCC, Sox2 expression is a controversial marker considering that some studies have reported Sox2 to be linked to lymph node metastasis and poor survival, while others have found increased Sox2 expression to improve prognosis [12, 13].

L1CAM is a neuronal cell adhesion molecule that has been studied mainly for its role in the nervous system. Following its role in cell motility and plasticity, L1CAM has been studied in multiple cancers and is considered a negative prognostic factor in endometrial, ovarian, breast, gastric, colon, pancreatic, kidney, non-small cell lung cancer, and melanoma [14]. According to the available literature on PubMed, only one study has investigated the role of L1CAM in OSCC and found that it was correlated with poor histologic differentiation and higher invasion [15]. However, no studies have correlated the expression of L1CAM with the survival of OSCC patients.

The objective of this study was to evaluate the protein expression of CD44, CD133, L1CAM, and SOX2 and correlate their expression with risk habits, clinicopathologic factors, and overall and disease-free survival in a high-risk, resource-constrained oral cavity cancer population.

## 2. Materials and Methods

The Aga Khan University Hospital (AKUH) is a Joint Commission International (JCI), and College of American Pathologists (CAP) accredited largest tertiary-care academic medical centre situated in Karachi. It serves as the preferred referral centre for cancer patients of all socioeconomic backgrounds from all over the country.

Patients had consented to participation and had complete clinicopathological information. All patient information was retrieved from the hospital's medical records and clinic follow-ups. The minimum follow-up time for all patients was 60 months. Overall survival (OS) was taken as the number of months from date of diagnosis until last known status (if alive) or date of death. Disease-free survival (DFS) was taken as the number of months from the date of surgery until recurrence or if no recurrence then until the last follow-up (if alive) or death. Ethical approval was obtained from the Ethical Review Committee of AKUH (ERC# 2020-0392-14105).

### 2.1. Sample Size Calculation

This was a retrospective cohort study comprising 100 OSCC patients who had been diagnosed and treated in the years January 1991–December 2015. The sample size calculation for this study was performed on Open Epi software (https://www.openepi.com/SampleSize/SSCohort.htm). According to the calculations, a sample size of 100 was deemed sufficient. An anticipated frequency of expression of CSC markers among OSCC patients ranging from 10.2% for CD44, 5.8% for CD133, and 7% for SOX2 [16–18] was used with a 90% level of significance, 5% precision, and design effect of 1.

### 2.2. Immunohistochemistry Performance

Before immunohistochemistry (IHC) performance, haematoxylin and eosin (H&E) stained slides of all tumour specimens were reviewed to confirm tumour content and tissue adequacy. IHC was performed manually. Formalin-fixed paraffin-embedded (FFPE) blocks were sectioned using a semiautomatic rotary microtome (pfm Rotary 3005E, pfm medical, Germany). Four-micrometre-thick tissue sections were transferred to a floating water bath to remove wrinkles and taken onto glass slides (FLEX IHC Microscope Slides, K8020, Dako, Denmark). Deparaffinization was performed for 30 min at 56°C in an oven, followed by dipping in xylene for 2 min. Slides were then rehydrated using water-ethanol serial dilutions (100%, 90%, 70%, and 50%) with a final rinse in deionized water. The EnVision FLEX, High pH (Link) system (K8000221, Dako, Denmark) was used for IHC staining according to the manufacturer's recommendations. To unmask the antigen of interest, target retrieval was performed by immersing slides in high pH target retrieval solution (K8004, Dako, Denmark) for 30 min in a water bath heated at 90–95°C. Following retrieval, slides were dipped in peroxidase blocking reagent (S2023, Dako, Denmark) to inhibit the activity of endogenous peroxidase. Following each step, slides were washed with Tris buffer saline + Tween 20 (wash buffer, S3006, Dako, Denmark). Sections were incubated in the primary antibody (CD44, CD133, L1CAM, and SOX2) according to their respective conditions.  Table 1 lists the primary antibody information including clone, company, dilutions, and incubation times. The primary antibody was rinsed off with wash buffer, and the slides were treated with secondary antibody EnVision/HRP (labelled-polymer rabbit/mouse, Dako, Denmark) and incubated for another 30 min. To visualize the antigen-antibody conjugate, DAB + chromogen (Dako, Denmark) was applied for 4 min and slides were dipped in haematoxylin (CS70030, Dako, Denmark) for 30 s for counterstaining. Specimens were dehydrated in a water-ethanol graded series (50%, 70%, 90%, and 100%) and mounted with cover slides using toluene-free mounting medium (Dako, Denmark). Experimental controls were run in each batch. A previously known positive specimen for each antibody (according to the manufacturer's recommendation) was selected (Table 1) as positive control, and a slide stained with saline instead of primary antibody served as the negative control.

### 2.3. Immunohistochemistry Evaluation and Scoring

Slides were observed under a light microscope (Nikon, Japan). Two independent observers (SMAA and RI) blinded to the patient history scored the slides. At least 200 cells in 5–10 different fields using a 20x lens were observed prior to scoring. The selection of the first field was subjective, while the remaining fields were selected systematically to cover the entire tumour specimen. A scoping view of the entire slide was taken at first glance, and the areas with the highest staining were selected for review as the first field. Following this, the slide was first observed in a horizontal manner and then in a vertical manner to observe the entire specimen and then assign scoring. The scoring of immunopositive expression was performed as summarized in Table 2.

### 2.4. Statistical Analysis

Statistical analysis was performed using Statistical Package for Social Sciences (SPSS) version 19 (IBM, USA). The expression of CD44, CD133, L1CAM, and SOX2 were correlated with patient demographics, clinical, pathological, and survival data. Patients were considered censored observation if they were alive at the time of last follow-up (for OS analysis) or were disease-free (for DFS analysis). Kaplan–Meier curves were drawn for OS and DFS analysis and compared using log-rank statistics. Cross-tabulations and logistic regression were run to correlate factors with markers expression and compared using the chi-square test or Fisher's exact test as appropriate. Odds ratios (OR) were reported with a 95% confidence interval (CI). Univariate Cox regression analysis was performed to evaluate the effect of markers expression and other factors on OS and DFS. Hazard ratios (HR) as estimates of relative risk were reported with 95% CI. All *P* values were two-sided and significant if <0.05.

## 3. Results

### 3.1. Patient Characteristics

The study cohort comprised 57 males and 43 females with a female:male ratio of 1:1.33. The mean age of patients was 51.42, SD ± 13.33, while the median age was 50 years. Eighty-two patients were ≥40 years of age, while ages for all participants ranged from 20 to 78 years. All patients underwent surgery for primary tumour resection. Some patients received additional treatment in the form of chemotherapy (8%), radiotherapy (65%), or palliative care (4%). Complete patient characteristics are available in Table 3.

### 3.2. CD44 Expression

**Figure 1:**
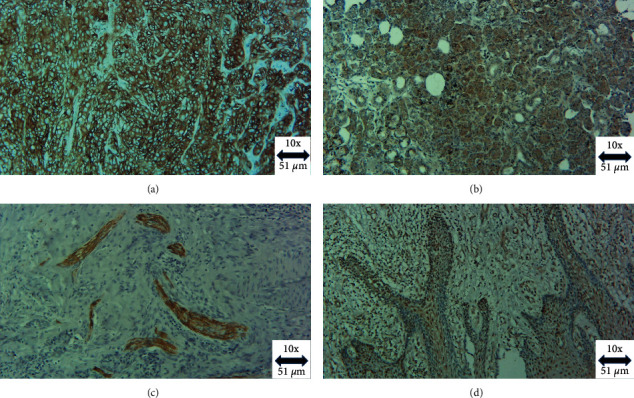
Photomicrograph of (a) CD44 cell membranous positivity, (b) CD133 cell membranous and cytoplasmic positivity, (c) L1CAM cell membranous positivity, and (d) SOX2 nuclear positivity in OSCC (magnification × 10).

CD44 immunohistochemical expression was observed as dark brown exclusively membranous staining (Figure 1(a)). CD44 positive expression was observed in the tumour cores of all patients and in the basal layer, which was expected since most epithelial stem cells are in the basal layer of the oral mucosal lining. CD44 expression was increased in the invasive front of the tissue and was present in all poorly differentiated tumours. High CD44 expression was seen in 33% of specimens, while the remaining 67% were classified as low CD44 expression. Although a greater number of patients with low CD44 expression were ≥40 years of age, this difference was not statistically significant (*P*=0.09).

Upon correlation of CD44 protein expression with patient clinicopathologic characteristics, it was seen that CD44-high patients had significantly advanced American Joint Committee on Cancer (AJCC) stage and T stage tumours (Table 4). Patients that were AJCC stage III had high CD44 expression (*P*=0.036) as well as those with tumour size T3 (*P*=0.007). Curiously, CD44 expression was also higher in patients that had floor of the mouth (71%) as a secondary site of tumour (*P*=0.038).

### 3.3. CD133 Expression

Cell membranous and cytoplasmic dark brown staining was seen in CD133 positive specimens (Figure 1(b)). CD133 positivity was observed in the plasma membrane protrusions in the tumour core cells and on the invasive front. There were 23 specimens positive for CD133 expression, while 77 were negative. Out of the 23 positive samples, 2 (9%) had a strong expression; 6 (26%) had moderate; and 15 (65%) had mild expression. A large group of patients ≥40 years of age tested negative for CD133 expression, but this did not translate to statistical significance (*P*=0.077).

An interesting observation was that chewing/smoking habits and the nature of habits were significant predictors of CD133 expression (Table 4). Patients who were habitual users (71%) had notably absent CD133 expression in comparison to non-users (*P*=0.003). The type of risk factor habit also appeared to affect CD133 expression as 69% of betel quid/areca nut users (*P*=0.015) and 65% of chalia/gutka/niswar users (*P*=0.047) had tumours that did not express CD133.

Furthermore, it was seen that CD133 expression was appreciably negative in tumours with a floor of mouth involvement (*P*=0.047). Contrarily, tumours that involved the tonsils had a 100% CD133 expression rate (*P*=0.051).

### 3.4. L1CAM Expression

Positive L1CAM expression was observed as diffuse patches of dark brown membranous staining in all cases, while in some patients, it was also present on the infiltration border of the tissue (Figure 1(c)). L1CAM positivity was seen in 41 specimens, while 59 were negative for L1CAM. The positive specimens were further classified as 34 (83%) mild, 5 (12%) moderate and 2 (5%) strong. Despite the high number of positive specimens observed L1CAM immunoexpression was not significantly affected by any of the clinicopathologic parameters or biomarkers tested (Table 4).

### 3.5. SOX2 Expression

Specimens positive for SOX2 expression exhibited dark brown nuclear staining (Figure 1(d)). SOX2 expression was observed in differentiated and less differentiated tissue layers alike, including the stratum basale and tumour cells resembling a basal-like phenotype. Total specimens positive for SOX2 expression were 63, while 37 did not express SOX2. The positive specimens included 32 (51%) mild, 28 (44%) moderate, and 3 (5%) strong. Although SOX2 was positive in many specimens, this did not translate into statistically significant interactions. It was seen that a large percentage of habitual smokers (71%) had positive SOX2 expression as compared to nonsmokers (*P*=0.086). Similarly, SOX2 expression was higher in moderately differentiated OSCC patients (71%), but this too was borderline significant (*P*=0.052).

### 3.6. Overall Survival (OS)

The survival rate in our patients at minimum 60 months follow-up was 44%. In Kaplan–Meier OS analysis, the median number of months for our patient cohort was 64. The median OS was higher in males versus females and in patients <40 years versus ≥40 years old; however, these differences were not significant. Similarly, the use of risk factors and primary tumour site was not significantly associated with survival. However, patients with subinvolvement of the tonsils had a significantly lower OS (*P* < 0.001, 9 vs. 100 months) than patients with no tonsils involved. Moreover, patients with positive neck pathology had a much shorter survival as compared to patients with no lymph node involvement (*P*=0.001, 31 vs. 155 months). Equally, the involvement of multiple lymph nodes instead of single also contributed to a starkly lower OS (*P*=0.001, 59 vs. 12 months). Likewise, stage N2 patients had the lowest survival at 12 months, as compared to N0 (149 months) and N1 (31 months) stages (*P* < 0.001).

**Figure 2:**
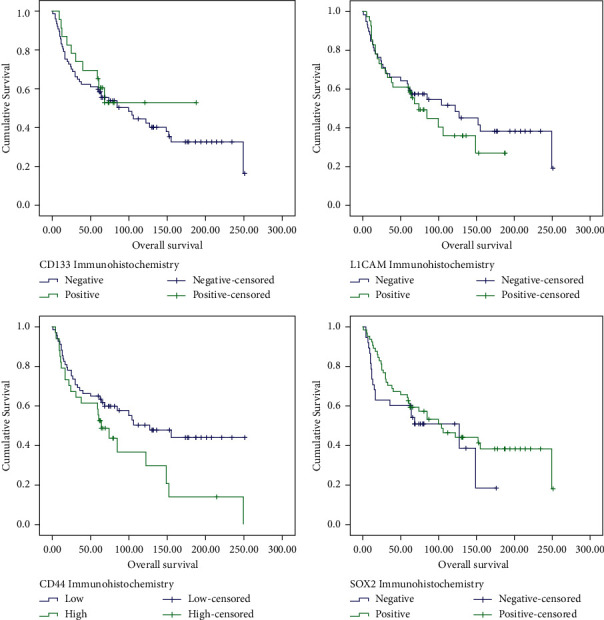
Kaplan–Meier curve analysis for overall survival of OSCC patients with *P*=0.613 for CD133 expression, *P*=0.047 for CD44 expression, *P*=0.489 for L1CAM expression, and *P*=0.318 for SOX2 expression.

The status of surgical margins was also a key predictor of OS as those with clear margins survived the longest at 149 months, and patients with involved margins had the worst median survival of only 13 months (*P*=0.004). The AJCC stage of patients was also a major prognostic indicator, as the survival of patients was highest for stage I patients (249 months) and was seen to steadily decrease with increasing AJCC stage until reaching worse survival for stage IV (14 months; *P*=0.002). In patients that received radiotherapy treatment, it was observed to significantly improve OS (*P*=0.026). Regarding biomarkers, patients with high CD44 expression had a significantly lower median OS at 64 months compared to 106 months for patients with low CD44 expression (*P*=0.047; Figure 2). Complete overall survival statistics are given in Table 5.

In Cox regression univariate analysis, the following factors were associated with a higher risk of death: tonsil involvement (*P*=0.004, HR = 8.99), involved primary margins (*P*=0.003, HR = 3.09), T4 tumour size (*P*=0.027, HR = 2.8), N2 stage (*P* < 0.001, HR = 4.15), AJCC stage IV (*P*=0.002, HR = 4.26), and radiotherapy received (*P*=0.029, HR = 1.95). Although CD44 was a significant predictor on Kaplan–Meier analysis, borderline significance was observed for CD44 expression in Cox regression analysis with *P*=0.051 and HR = 1.71. All other factors tested in univariate analysis are summarized in Table 6.

### 3.7. Disease-Free Survival (DFS)

**Figure 3:**
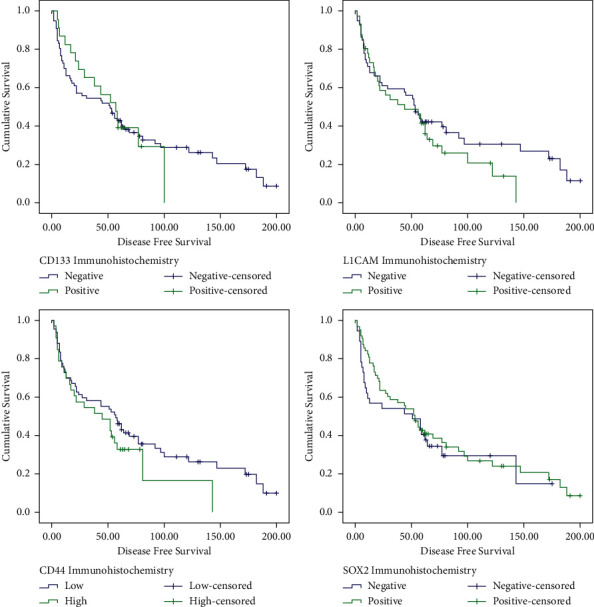
Kaplan–Meier curve analysis for disease-free survival of OSCC patients with *P*=0.996 for CD133 expression, *P*=0.24 for CD44 expression, *P*=0.266 for L1CAM expression, and *P*=0.483 for SOX2 expression.

The rate of recurrence observed in our OSCC patients at minimum 60 months follow-up was 74%. In Kaplan–Meier DFS analysis, the median months for recurrence were 52.5. Factors that were significant predictors of worse OS were also seen to predict worse DFS such as: tonsil involvement (*P*=0.001), neck pathology (*P*=0.018), involved primary margins (*P*=0.008), N2 stage (*P*=0.024), and AJCC stage IV (*P*=0.03). Additionally, cheek as primary tumour site (*P*=0.045) and skin involvement (*P*=0.031) were also seen to cause significantly lower median DFS months (Figure 3). For complete disease-free survival statistics, see Table 5.

In Cox regression univariate analysis, the following factors were associated with increased risk of recurrence: primary tumour site (*P*=0.049, HR = 1.66), tonsil involvement (*P*=0.007, HR = 7.69), skin involvement (*P*=0.045, HR = 3.32), primary margins being involved (*P*=0.004, HR = 2.756), T4 tumour size (*P*=0.034, HR = 2.28), N2 stage (*P*=0.025, HR = 2.36), and AJCC stage IV (*P*=0.009, HR = 2.64). Table 6 lists all factors tested for univariate DFS survival.

## 4. Discussion

Oral cancer is a heterogeneous disease, arising from the dysfunction of several molecular pathways, resulting in severe morbidity and oftentimes mortality. The survival of OSCC patients has remained largely unchanged for the past 40 years [19]. CSCs represent a group of markers that may be used to successfully estimate prognosis and serve as targets for molecular therapy, as CSC markers are mainly expressed in the basal layers of the oral mucosal surfaces and have frequently dysregulated expression in OSCC.

High CD44 expression was recently observed to be an independent predictor for prognosis in a study of 44 patients by Hendawy and Esmail [7]. The authors found that CD44 was increased in patients with advanced TNM stage and that it led to reduced DFS and 3-year OS. Although we found a lesser positivity percentage (33%) as compared to Esmail et al.'s (59%), the negative impact on overall survival was noted in both studies. Although CD44 led to a poor prognosis, a correlation with DFS was not determined in this cohort. This is similar to the conclusions of another study that found abundant CD44 expression in stage I and II OSCC cells but no correlation with disease recurrence [20]. However, another study group determined reduced DFS for CD44 positive patients [7]. The difference in positive cases can be attributed to the dissimilar genetic makeup of the populations under study, Egyptian and Pakistani, though the same antibody clone and similar scoring criteria were applied in both studies.

It is hypothesized that CD44 affects patient survival by conferring radio- and chemoresistance in the tumours and causing relapse and metastasis. Moreover, CD44 stimulates pathways that initiate and promote tumour cell proliferation and epithelial-to-mesenchymal transition [21]. This seems to be the case in this study as participants had advanced disease and moderately differentiated carcinomas.

The exact location of CD44 staining is also thought to influence prognosis. Boxberg et al. [22] compared the expression of CD44 within the tumour core, at the invasive margin, and in lymph node metastases; the invasive margin had the highest expression of all sites (39%) and was an independent predictor for worse survival and recurrence. On the other hand, Cohen et al. [23] studied a diverse population of black and Hispanic ethnicities and found that universal gross staining rather than peripheral staining was associated with poor overall survival. As they found a relatively high positivity of 62.5% in 40 specimens, it was concluded that the percentage of cells expressing CD44 was more influential on prognosis as compared to staining intensity or localization. This is also reflected in current study results as 33% CD44 universal staining led to worse patient survival.

An interesting observation in our data set was a significant number of the floor of the mouth tumours having high CD44 positivity. This was also noted by Krump and Ehrmann [24] who found a total of 62% positive specimens and significantly increased CD44 expression in the floor of the mouth tumours as compared to the tongue. This leads to the conclusion that the prognostic value of CD44 depends not only on the total expression in tumour but also on tumour location and maybe even on subcellular location.

Moreover, Hendawy [7] also found markedly higher CD44 expression in tumours of bigger size, overall higher TNM stage, lymphovascular invasion, and metastasis. Although similar correlations of CD44 with advanced T and AJCC stage were seen in this study, no effect of CD44 immunoexpression was observed on nodal involvement. Furthermore, no patients included in this study had metastasis, due to which comparisons cannot be drawn.

In the case of CD133, 23% positivity was observed with the majority (15/23) being mild positive. Other groups investigating CD133 expression have reported wide-ranging figures including 5.8% [17], 68% [25], and even 100% positivity [26].

There were unremarkable survival differences among the CD133+ and CD133– patient groups. Similarly, several other groups investigating CD133 expression in the oral cavity found no associations either patient characteristics or survival [17, 25, 26]. On the other hand, the progression of oral potentially malignant disorders to squamous cell carcinoma has been linked to high CD133 expression in premalignant specimens [27, 28]. It is hypothesized that CD133 may play a role in initiating malignancy in early stages and cease to be a key regulator once carcinoma has fully developed. Since the patients of this study all had fully developed and advanced OSCC, the role of CD133 was not prominently observed.

Another observation was that patients who were habitual chewers of oral cancer risk products such as areca nut, betel quid, smoking and smokeless tobacco, and so on were more prone to having CD133– tumours. As per the author's knowledge, this has not been reported before. This may be explained by the fact that patients with chewing habits develop usually potentially malignant conditions, and some authors have found that CD133-cell populations may be more tumourigenic than CD133+ cells [29], ultimately causing the patients to undergo malignant transformation. In our team's experience, patients continued their addictive risk factor habits even during and immediately after treatment, despite regular counselling. Due to these prevalent habits, the genetic makeup of OSCC in Pakistani patients is bound to differ from Western literature. Although other CSC markers were investigated in association with betel chewing in the population of Taiwan and Sri Lanka, but no significant effect of risk factor habits on markers expression was seen [30, 31].

Furthermore, 63% positivity was observed for SOX2 in the present study, while previous reports have varied widely with as low as 7% [18] and as high as 100% [13] reported SOX2 positivity.

High SOX2 protein expression was observed in inpatients with moderately differentiated OSCC, but this was borderline significant (*P*=0.052). These may be etiologic findings since a meta-analysis of SOX2 expression in head and neck cancer found that high immunoexpression leads to worse five-year survival [32]. Contrarily, other authors have suggested smaller tumour size and improved DFS for SOX2 expressing tumours [12].

The differences in findings may be due to the highly variable thresholds for positivity that have been used and also the classifications of positive staining into diffuse and peripheral patterns, with the diffuse pattern exhibiting lymph node metastasis and poorer survival [13]. Furthermore, a study utilizing a rabbit polyclonal antibody and similar staining criteria as the present study found that SOX2 was involved more in the early tumourigenesis events rather than the progression of developed OSCCs [18]. They detected SOX2 overexpression as an independent predictor of malignant transformation for oral leucoplakia, while SOX2 expression in OSCC was associated with early T and N stages and better survival. Since our cohort did not include premalignant conditions, these findings were not reproduced.

Regarding L1CAM, as per our understanding, this is the first time that L1CAM immunoexpression was correlated with survival in OSCC. Since 41% of tumours were positive for L1CAM, it cannot be ruled out as a CSC marker for OSCC. Although previous findings indicate that increased L1CAM expression leads to poor histologic differentiation [15], these were not replicated in the present study cohort as L1CAM positivity was roughly inversely proportional to histological differentiation. However, the sample size in the cited study was only 25 OSCCs, while we studied 100 OSCCs and found no such association. Moreover, the percentage positivity of L1CAM and scoring criteria used was also not fully elaborated in the above-cited study.

In this group, the rate of survival was significantly lower in patients who suffered recurrence as compared to those who did not: patients with recurrence had 38 times higher risk of death. It was reported by Camisasca et al. [33] that the 5-year survival rate was 3 times lower in patients with recurrence than those without. Several other reports have assessed the effect of patient clinicopathologic factors on survival, and in line with the majority of studies, we found conventional and established prognostic indicators, such as involved lymph nodes, higher AJCC and TNM stages, and involved surgical margins, were all significantly associated with OS and DFS in this patient cohort [34].

Over the past decade, hundreds of biomarkers for OSCC have been studied in numerous studies, but none of them has been adopted into clinical practice. This is often due to small sample sizes, inadequate validation of the marker using multiple techniques, and dearth of prospective studies. Nevertheless, the present study adds unique insights to our understanding of oral cancer using a panel of CSC markers on the same well-characterized cohort from a resource-constrained high-risk population. The present work sheds light on a population that is at high risk for oral cancer and ironically is much less studied due to limited scientific resources. Cancer stem cell markers help identify a subset of the tumour population that is responsible for the bulk of tumour-related characteristics and resists conventional treatment. Once this subpopulation is identified, in the next step, it can be targeted so that this self-renewal of the tumour can be halted, and complete remission can be achieved.

## 5. Conclusion

The present study found that high CD44 protein expression correlated with adverse overall survival of OSCC patients. Moreover, increased CD44 immunoexpression was more common in patients with AJCC stage III and T3 tumours. On the other hand, CD133 was significantly lower in patients with chewing habits but did not ultimately change the prognosis. SOX2 and L1CAM were impartial for OS and DFS, while tonsils, nodal involvement, and AJCC stage were independent predictors of poor OS and DFS.

## Figures and Tables

**Table 1:** IHC protocol and antibody details.

S. no.	Antibody	Clone/product code	Source, clonality	Company	Antibody dilution	Antibody incubation	Positive control	Cellular location
1.	CD133	EPR16508	Rabbit, monoclonal	Abcam, UK	1 : 1,000	40 min	Glioblastoma multiforme	Cell membranous and cytoplasmic
2.	CD44	DF1485	Mouse, monoclonal	Dako, Denmark	1 : 40	40 min	Glioblastoma multiforme	Cell membranous
3.	L1CAM	EPR18750	Rabbit, monoclonal	Abcam, UK	1 : 500	40 min	Normal kidney	Cell membranous
4.	SOX2	ab97959	Rabbit, polyclonal	Abcam, UK	1 : 250	40 min	Glioblastoma multiforme	Nuclear

**Table 2:** Immunoreactivity scoring criteria for all antibodies.

Antibody	No. of positive cells	Type of staining	Scoring	For statistical analysis	References
CD133	0	—	Negative (0)	Negative	[35]
	<30%	—	Weak (+/1)	Positive	
	30–60%	—	Moderate (++/2)	Positive	
	>60%	—	Strong (+++/3)	Positive	
CD44	≤10% cells	Weakly stained	Negative (0)	Negative	[36]
	11–30% cells	Weakly stained	1 (low)	Low	
	>30% weakly or <30% moderately stained		2 (low)	Low	
	30%–60%	Moderately stained	3 (high)	High	
	>60%	Moderately or strongly stained	4 (high)	High	
L1CAM	0% = 0	None = 0	Intensity × % of positive cells = score		[37]
	<10% = 1	Weak = 1	0–2 = negative	Negative	
	10–50% = 2	Moderate = 2	3–4 = weakly positive	Positive	
	51–80% = 3	Strong = 3	6–8 = moderately positive	Positive	
	>80% = 4		9–12 = strong	Positive	
SOX2	<10%	—	Negative (0)	Negative	[38]
	10–50%	—	Weak (+/1)	Positive	
	50–90%	—	Moderate (++/2)	Positive	
	>90%	—	Strong (+++/3)	Positive	

**Table 3:** Patient characteristics (*n* = 100).

Characteristics	No.	Characteristics	No.
*Gender*		*AJCC stage*	
Male	57	Stage I	19
Female	43	Stage II	32
*Age division*		Stage III	23
<40 years	18	Stage IV	26
40 and >40 years	82	*Tonsil*	
*Habits*		Yes	2
Yes	79	No	98
No	21	*Skin involvement*	
*Habit pattern*		Yes	3
Single	37	No	97
Multiple	42	*Palate*	
Non-users	21	Yes	8
*Tobacco/smoking*		No	92
Yes	35	*Mandible*	
No	65	Yes	26
*Paan/supari*		No	74
Yes	61	*Retromandibular*	
No	39	Yes	16
*Chalia/gutka/niswar*		No	84
Yes	31	*Floor of mouth*	
No	69	Yes	7
*Primary tumour site*		No	93
Cheek	63	*Surgical margins*	
Tongue	37	Clear	62
*Histological differentiation*		Near	27
WDSSC	37	Involved	11
MDSCC	59	*Radiotherapy*	
PDSCC	4	Yes	65
*Size of primary tumour (T)*		No	35
T1	21	*Overall survival*	
T2	47	Alive	44
T3	15	Dead	56
T4	17	*Recurrence*	
*Lymph node metastasis (N)*		Yes	74
N0	77	No	26
N1	13	*SOX-2*	
N2	10	Positive	63
*CD44*		Negative	37
High	33	*SOX-2 staining intensity*	
Low	67	Mild	32
*CD133*		Moderate	28
Positive	23	Strong	3
Negative	77	*L1CAM*	
*CD133 staining intensity*		Positive	41
Mild	15	Negative	59
Moderate	6	*L1CAM staining intensity*	
Strong	2	Mild	34
		Moderate	5
		Strong	2

**Table 4:** Correlations of antibody expression and patient characteristics.

Clinicopathologic parameters	Total cases	CD133			CD44			L1CAM			SOX2		
		−ve	+ve	*P*	Low	High	*P*	−ve	+ve	*P*	−ve	+ve	*P*
*Age*													
<40 years	18	11	7	0.077	9	9	0.090	12	6	0.465	6	12	0.722
≥40 years	82	66	16		58	24		47	35		31	51	
*Gender*													
Male	57	41	16	0.165	38	19	0.935	30	27	0.136	19	38	0.382
Female	43	36	7		29	14		29	14		18	25	
*Habits*													
Yes	79	56	23	**0.003** ^**∗**^	54	25	0.576	46	33	0.761	30	49	0.695
No	21	21	0		13	8		13	8		7	14	
*Betel quid/areca nut*													
Yes	61	42	19	**0.015** ^**∗**^	43	18	0.353	37	24	0.674	22	39	0.809
No	39	35	4		24	15		22	17		15	24	
*Smoking/tobacco use*													
Yes	35	25	10	0.331	23	12	0.841	19	16	0.482	9	26	0.086
No	65	52	13		44	21		40	25		28	37	
*Chalia/gutka/naswar*													
Yes	31	20	11	**0.047** ^**∗**^	20	11	0.723	16	15	0.314	15	16	0.114
No	69	57	12		47	22		43	26		22	47	
*Habit pattern*													
Single	37	27	10	0.929	25	12	0.848	22	15	0.934	16	21	0.613
Multiple	42	29	13	0.998	29	13	0.663	24	18	0.855	14	28	0.460
Non-users	21	21	0	0.998	13	8	0.571	13	8	0.718	7	14	1
*Primary tumour site*													
Cheek	63	48	15	0.802	41	22	0.594	36	27	0.622	22	41	0.574
Tongue	37	29	8		26	11		23	14		15	22	
*Palate*													
Yes	8	6	2	1	6	2	1	5	3	1	3	5	1
No	92	71	21		61	31		54	38		34	58	
*Mandible*													
Yes	26	22	4	0.283	15	11	0.241	14	12	0.535	10	16	0.858
No	74	55	19		52	22		45	29		27	47	
*Floor of the mouth*													
Yes	7	3	4	**0.047** ^**∗**^	2	5	**0.038** ^**∗**^	2	5	0.119	3	4	0.708
No	93	74	19		65	28		57	36		34	59	
*Tonsils*													
Yes	2	0	2	0.051	0	2	0.107	0	2	0.166	1	1	1
No	98	77	21		67	31		59	39		36	62	
*Skin*													
Yes	3	3	0	1	2	1	1	1	2	0.566	0	3	0.294
No	97	74	23		65	32		58	39		37	60	
*Differentiation*													
Well differentiated	37	25	12	0.313	26	11	0.790	21	16	0.785	18	19	0.131
Moderately differentiated	59	48	11	0.127	38	21	0.554	35	24	0.804	17	42	0.052
Poorly differentiated	4	4	0	0.999	3	1	0.844	3	1	0.491	2	2	0.959
*Primary margins*													
Clear	62	48	14	0.938	42	20	0.964	36	26	0.476	23	39	0.783
Near	27	21	6	0.970	18	9	0.921	18	9	0.446	9	18	0.734
Involved	11	8	3	0.735	7	4	0.790	5	6	0.440	5	6	0.600
*T classification*													
T1	21	17	4	0.461	18	3	0.058	14	7	0.636	8	13	0.849
T2	47	33	14	0.358	32	15	0.138	27	20	0.474	16	31	0.747
T3	15	12	3	0.943	6	9	**0.007** ^**∗**^	7	8	0.234	7	8	0.608
T4	17	15	2	0.544	11	6	0.140	11	6	0.899	6	11	0.859
*N classification*													
N0	77	60	17	0.856	54	23	0.413	46	31	0.921	27	50	0.732
N1	13	10	3	0.936	8	5	0.538	7	6	0.690	6	7	0.445
N2	10	7	3	0.577	5	5	0.209	6	4	0.987	4	6	0.759
*AJCC clinical stage*													
I	19	15	4	0.692	16	3	0.157	13	6	0.569	8	11	0.768
II	32	23	9	0.576	23	9	0.321	17	15	0.286	10	22	0.434
III	23	17	6	0.703	12	11	**0.036** ^**∗**^	12	11	0.288	10	13	0.929
IV	26	22	4	0.624	16	10	0.107	17	9	0.831	9	17	0.609
*Radiotherapy*													
Yes	65	49	16	0.601	41	24	0.256	41	24	0.259	22	43	0.373
No	25	28	7		26	9		18	11		15	20	
*CD133*													
Positive	23	—	—	—	15	8	0.836	10	13	0.850	9	14	0.809
Negative	77	—	—		52	25		49	28		28	49	
*CD44*													
High	33	25	8	0.836	—	—	—	20	13	0.819	14	19	0.43
Low	67	52	15		—	—		39	28		23	44	
*L1CAM*													
Positive	41	28	13	0.085	28	13	0.819	—	—	—	17	24	0.441
Negative	59	49	10		39	20		—	—		20	39	
*SOX2*													
Positive	63	49	14	0.809	44	19	0.43	39	24	0.441	—	—	—
Negative	37	28	9		23	14		20	17		—	—	

^*∗*^ *P* < 0.05 taken as significant.

**Table 5:** Kaplan–Meier (log-rank statistic) analysis for overall survival and disease-free survival (*n* = 100).

Variable	Total	OS in months	*P*	95% CI		DFS in months	*P*	95% CI	
		Median		Lower	Upper	Median		Lower	Upper
*Gender*									
Male	57	100	0.597	37.7	162.3	51	0.629	28.1	73.9
Female	43	85		30.1	139.4	58		35.4	80.6
*Age division*									
<40 years	18	155	0.93			31	0.376	0	80.9
>40 years	82	100		60.2	139.8	52		35.1	69
*Primary tumour site*									
Cheek	63	85	0.287	46.6	123.4	44	**0.045** ^**∗**^	21.6	66.4
Tongue	37	155				58		20.1	95.8
*Tonsil*									
Yes	2	9	**<0.001** ^**∗**^			5	**0.001** ^**∗**^		
No	98	100		53.1	146.9	53		40.8	65.2
*Skin*									
Yes	3	25	0.082	4.2	45.8	7	**0.031** ^**∗**^	2.2	11.9
No	97	104		54.6	153.5	53		37.1	68.9
*Neck pathology*									
Positive	27	31	**0.001** ^**∗**^	0	102.2	69	**0.018** ^**∗**^	51.8	86.2
Negative	51	155				22		0	52.5
ND	22	64		13	115	27		4	50
*Pathologically involved lymph nodes*									
Single	16	59	**0.001** ^**∗**^	2.2	115.8	29	0.078	0	85.8
Multiple	11	12		6.6	17.4	6		2.8	9.2
NA	73	149		85.4	212.6	58		40.9	75.1
*Primary margins*									
Clear	62	149	**0.004** ^**∗**^	88.5	209.5	62	**0.008** ^**∗**^	49	75
Near	27	62		23.2	100.8	24		0	76.6
Involved	11	13		9.8	16.2	7		0	15.6
*N classification*									
N0	77	149	**<0.001** ^**∗**^	79.2	218.8	58	**0.024** ^**∗**^	48.6	67.4
N1	13	31		11.6	50.4	27		5.9	48.1
N2	10	12		9	15	6		2.9	9.1
*AJCC stage*									
I	19	249	**0.002** ^**∗**^	34.7	463.3	69	**0.03** ^**∗**^	51.8	86.3
II	32	149		82.5	215.5	58		39.7	76.3
III	23	68		47.5	88.5	51		22.8	79.2
IV	26	14		0	30.2	9		2	16
*Radiotherapy*									
Yes	65	68	**0.026** ^**∗**^	43.5	92.5	44	0.242	18.3	69.7
No	35					62		35	89
*CD44*									
High	33	64	**0.047** ^**∗**^	46.8	81.2	45	0.24	11.2	78.8
Low	67	106		39.9	172.1	57		39.3	74.7
*CD133*									
Positive	23		0.613			57	0.996	35.1	79
Negative	77	100		55.5	144.5	52		24.5	79.6
*L1CAM*									
Positive	41	74	0.489	42.7	105.3	44	0.266	5.1	82.9
Negative	59	122		42.4	201.6	53		40.2	65.8
*SOX2*									
Positive	63	100	0.318	53.7	146.3	53	0.483	39.8	66.2
Negative	37	68			136.4	51		0	104.6

^*∗*^ *P* < 0.05 taken as significant.

**Table 6:** Cox regression univariate analysis (*n* = 100).

Characteristic	Overall survival				Disease-free survival			
	*P*	Hazard ratio	95% CI		*P*	Hazard ratio	95% CI	
		HR	Lower	Upper		HR	Lower	Upper
*Gender*		100				100		
Male		1.0 (ref)				1.0 (ref)		
Female	0.598	1.154	0.678	1.962	0.632	0.893	0.562	1.419
*Age division*		100				100		
< 40 Years		1.0 (ref)				1.0 (ref)		
> 40 Years	0.931	0.969	0.472	1.987	0.381	0.769	0.427	1.385
*Primary tumour site*		100				100		
Tongue		1.0 (ref)				1.0 (ref)		
Cheek	0.291	0.738	0.419	1.297	**0.049** ^**∗**^	1.655	1.002	2.734
*Tonsil*		100				100		
No		1.0 (ref)				1.0 (ref)		
Yes	**0.004** ^**∗**^	8.999	2.016	40.167	**0.007** ^**∗**^	7.691	1.731	34.18
*Skin*		100				100		
No		1.0 (ref)				1.0 (ref)		
Yes	0.097	2.699	0.836	8.718	**0.045** ^**∗**^	3.316	1.029	10.69
*Primary margins*		100				100		
Clear	**0.007** ^**∗**^	1.0 (ref)			0.11	1.0 (ref)		
Near	**0.045** ^**∗**^	1.856	1.014	3.396	0.109	1.535	0.909	2.594
Involved	**0.003** ^**∗**^	3.091	1.454	6.572	**0.004** ^**∗**^	2.756	1.372	5.537
*T classification*		100				100		
T1	0.135	1.0 (ref)			0.156	1.0 (ref)		
T2	0.215	1.655	0.746	3.675	0.39	1.324	0.698	2.512
T3	0.085	2.23	0.896	5.548	0.162	1.732	0.802	3.743
T4	**0.027** ^**∗**^	2.803	1.124	6.991	**0.034** ^**∗**^	2.28	1.066	4.874
*N classification*		100				100		
N0	**<0.001** ^**∗**^	1.0 (ref)			**0.031** ^**∗**^	1.0 (ref)		
N1	**0.004** ^**∗**^	2.888	1.415	5.89	0.082	1.833	0.925	3.635
N2	**<0.001** ^**∗**^	4.148	1.89	9.103	**0.025** ^**∗**^	2.364	1.114	5.018
*AJCC stage*		100				100		
I	**0.004** ^**∗**^	1.0 (ref)			**0.038** ^**∗**^	1.0 (ref)		
II	0.345	1.579	0.612	4.075	0.436	1.329	0.65	2.717
III	**0.033** ^**∗**^	**2.789**	1.088	7.129	0.143	1.755	0.827	3.726
IV	**0.002** ^**∗**^	4.259	1.681	10.792	**0.009** ^**∗**^	2.644	1.269	5.512
*Radiotherapy*		100				100		
Yes		1.0 (ref)				1.0 (ref)		
No	**0.029** ^**∗**^	1.950	1.071	3.552	0.247	0.749	0.459	1.222
*CD44*		100				100		
Low		1.0 (ref)				1.0 (ref)		
High	**0.051**	1.71	0.999	2.927	0.246	1.346	0.815	2.223
*CD133*		100				100		
Negative		1.0 (ref)				1.0 (ref)		
Positive	0.615	0.837	0.418	1.676	0.996	0.998	0.568	1.756
*L1CAM*		100				100		
Negative		1.0 (ref)				1.0 (ref)		
Positive	0.491	1.208	0.706	2.066	0.272	1.304	0.812	2.092
*SOX2*		100				100		
Negative		1.0 (ref)				1.0 (ref)		
Positive	0.321	1.247	0.668	2.325	0.265	1.364	0.79	2.353

^*∗*^ *P* < 0.05 taken as significant.

## Data Availability

The patient data used to support the findings of this study are included within the article.
